# Cultivating quality awareness in corona times

**DOI:** 10.1007/s11019-021-10010-x

**Published:** 2021-03-31

**Authors:** Guus Timmerman, Andries Baart, Jan den Bakker

**Affiliations:** 1 Presence Foundation, Grebbeberglaan 15, 3527 VX Utrecht, The Netherlands; 2 Optentia Research Focus Area, North-West University, Vanderbijlpark, South Africa; 3 University Medical Center Utrecht, Heidelberglaan 100, 3584 CX Utrecht, The Netherlands

## Abstract

The Covid-19 pandemic is a tragedy for those who have been hard hit worldwide. At the same time, it is also a test of concepts and practices of what good care is and requires, and how quality of care can be accounted for. In this paper, we present our Care-Ethical Model of Quality Enquiry (CEMQUE) and apply it to the case of residential care for older people in the Netherlands during the Covid-19 pandemic. Instead of thinking about care in healthcare and social welfare as a set of separate care acts, we think about care as a complex practice of relational caring, crossed by other practices. Instead of thinking about professional caregivers as functionaries obeying external rules, we think about them as practically wise professionals. Instead of thinking about developing external quality criteria and systems, we think about cultivating (self-)reflective quality awareness. Instead of abstracting from societal forces that make care possible but also limit it, we acknowledge them and find ways to deal with them. Based on these critical insights, the CEMQUE model can be helpful to describe, interrogate, evaluate, and improve existing care practices. It has four entries: (i) the care receiver considered from their humanness, (ii) the caregiver considered from their solicitude, (iii) the care facility considered from its habitability and (iv) the societal, institutional and scholarly context considered from the perspective of the good life, justice and decency. The crux is enabling all these different entries with all their different aspects to be taken into account. In Corona times this turns out to be more crucial than ever.

The Corona virus disease 2019 (Covid-19) pandemic is a tragedy for those who have been hard hit worldwide. At the time of writing the original manuscript, the total number of confirmed cases worldwide was above 5.6 million. More than 350 thousand people had died. At the time of revising the manuscript, seven months later, those numbers have increased to more than 90 million confirmed cases and more than 1.9 million deaths.^1^ Many people are experiencing difficult times, not only physically but also mentally and socially. Large numbers, not suffering from Covid-19 itself, are also experiencing hardship because of the measures taken, measures that make them unemployed, kill their businesses, isolate them from relatives and leave them without the care they need. That is the case in Europe and the USA, but even more so in poverty-stricken and bombed Yemen, the overcrowded favelas of Rio de Janeiro, the slums of Mumbai or the townships of South Africa. At the same time, the Covid-19 pandemic is also a test of our concepts and practices of what good care is and what it requires, and how quality of care can be accounted for. However understandable, it is also significant that the moment the pandemic arrived in the Netherlands the Healthcare and Youth Inspectorate temporarily took its own quality system out of operation. It raises the question of what this system actually serves. In this article, we put our Care-Ethical Model of Quality Enquiry (CEMQUE) to the test^2^.

## Assessing quality of care

After decades of research into a range of care practices in healthcare and social welfare, we have become convinced that good care requires relational caring. For many years now we have been publishing on relational caring and providing training and supervision for (teams of) care professionals (Baart 2001; Baart et al. 2011; Baart and Vosman 2011, 2015). Over time we have come to realise that, however helpful training and supervision may be, they make little difference when professionals are evaluated, judged and rewarded by quality systems that are not equipped to perceive, recognise and acknowledge relational caring. This has led us to develop a new way of thinking about quality that is able to do justice to relational caring, as well as a model to guide inquiry into and critically determine the quality of care received. A way of thinking that includes not only a perspective ‘from nowhere’ (Jerak-Zuiderent 2015) but also perspectives from within; not only ‘facts and figures’ (abstracted reality) but also phenomenology (lived reality), narrativity (signified reality), participation (shared reality) and deliberation (evaluated reality). This thinking and the resulting CEMQUE model are based on more than ten years of research, study and deliberation with caring professionals and their organisations (Baart 2004b, 2008, 2014, 2018; Baart and Grypdonck 2008; Baart 2018; Vosman and Baart 2011).

The wider, normative context of our thinking is a political take on care ethics as ‘an interdisciplinary approach (1) that understands care as an everyday practice that enables to live together, and therefore is eminently political (). (2) Caring practices are socially and institutionally embedded, care not being accessible in a free floating ‘essence’, not ontologically or even metaphysically determinable. (3) Relational interaction in care ethics is seen as both a locus of knowing (i.e. an epistemologically relevant as well as contested locus) and a locus of recognition and attention (). (4) Within the explicitly broad view on care *good* medicine, health care, help, and support are always context- and situation-specific, and can only be validated locally—that is, via the experience of the care receiver (). (5) Care ethics as we practice it, studies practices of medicine, health care and social welfare, help, and support, both empirically and theoretically and not just philosophically or via a general political theory only’ (Timmerman et al. 2019: 574; further references there).

In this paper, we present the CEMQUE model and apply it to the case of residential care for older people during the Covid-19 pandemic. In the Netherlands, the nursing homes were hard hit during the first wave of infections by the Sars-CoV-2 virus. The first confirmed case of Covid-19 in a nursing home was reported on March 12th, 2020. By the first week of April, approximately 40% of Dutch nursing homes reported infections. As the Netherlands was facing shortages of personal protection equipment and a lack of diagnostic capacities, the government issued a nationwide visiting ban for nursing homes on March 19th (Sizoo et al. 2020). The ban was implemented without question by the boards of organisations providing care for the elderly (den Uijl et al. 2020). It did not however prevent the virus from killing many residents. In some of the nursing homes up to one third of their population died. Infected residents with dementia were either isolated in their room or moved to a special Corona cohort department. These measures were advised by the Dutch national association of residential and home care organisations, ActiZ, and the Dutch association of elderly care physicians, Verenso. The consequences of the ban on visits were in many cases also devastating (van der Roest et al. 2020), including people dying while being left alone without understanding why their children were no longer visiting them (den Uijl et al. 2020). Evaluating this ban in the summer, the government decided not to issue a nationwide visiting ban again (den Uijl et al. 2020; Sizoo et al. 2020; Frederiks et al. 2020). In the second wave, starting in September, nursing homes again faced a rise in infections. Once again people with dementia have been isolated or moved to special Corona wings. One nursing home however, De Riethorst, decided to take a different path.^3^

**‘De Riethorst’ nursing home in the second wave of Corona infections**

The new building of De Riethorst nursing home opened its doors in September 2020. It was designed to give elderly residents with dementia more freedom to go wherever they want and more opportunity for contact with people without dementia. It was intended to become an example for the whole of the Netherlands, as the director explained in December 2017: “You won’t find this anywhere else yet. Our nursing home should be a home for the customer. Why the term customer? The customer is king and can decide for himself what he or she does. In a traditional nursing home, people just have to follow the rules of the house”.

At the end of November 2020, two of the 55 residents displayed mild symptoms and turned out to be infected with the Sars-CoV-2 virus. When other residents were tested, many of them turned out to be infected too. At first, the management and nursing home doctor decided to isolate infected residents in their rooms, in accordance with the guidelines of ActiZ and Verenso. As the number of infections rose, they realised what they were asking of their residents with dementia. As the nursing home physician later explained: “People with dementia do not understand why rules are imposed as they are. Or why they must suddenly stay in their room from one day to the next while they could move freely through the building the day before. You cannot explain it. And after a minute they have often forgotten again. This is also about quality of life. In Corona times too, it is important to consider quality of life because we do not know how long people will need to be isolated in their rooms. The dilemma is that there are no right choices. All choices are less than satisfactory, none are good, but you still have to choose. The dilemma is also that you cannot say: let me think about it for a week”.

So, De Riethorst decided to set up a Corona cohort department, also in accordance with the guidelines of ActiZ and Verenso. The cohort department can only be entered via a buffer zone and with personal protective equipment. But management and doctor also consulted residents’ families with the proposal not to isolate anyone and to leave every resident, whether infected or not, the freedom of movement they are used to. Families were given the choice between having their non-infected relative isolated or temporarily taking them home. The nursing home explains that she has known most of the families for some time, which makes it easier to confront them with this difficult decision. None of the families decided to take their family member home. Only a few chose to have them isolated. The other families apparently decided to run the risk of their family member becoming infected. After two weeks, almost all the residents were infected. Some of them died, others recovered. Only one family turned to the media to complain about being given too short a time to decide. The director regrets not having been able to contact this family in time but stands behind the decision: “The residents are not sick. They have a form of dementia. Should you sedate them to prevent them from wandering around? Then they may die because of that. At De Riethorst, clients can always leave their apartment because freedom is of paramount importance. Should we change that in a hurry?” She also realises that their decision is burdensome for the nurses and caregivers, especially because the public has become less tolerant about measures taken by the government and by care organisations.

The special thing about what De Riethorst did was that it took full account of what it was there for, and for whom it was there: people with advanced dementia in the last phase of their life. It decided to give residents freedom of movement; it realised what they could understand and handle, and what it was asking from them if they were isolated or moved. When facing ‘devilish dilemmas’, it did not turn away from what it perceived as its purpose, but instead let itself be led by it. De Riethorst also involved the families in their decision-making, making use of the relationships they had built up in the years before. And it was aware of its responsibility to be a hospitable organisation, also for its employees. The idea of good care held by the management and the physician of De Riethorst was much broader than safety, or simply preventing residents from becoming infected. Below, we show how the CEMQUE model can help to make this reasoning explicit, ask questions, show tensions and suggest different paths to take. First though, we introduce our thinking about good care and quality of care.

The usual method for determining whether care receivers receive good care is to ask them, or their relatives, afterwards: are you satisfied? They are systematically asked, for example by filling in a survey, for their patient or client experiences regarding different aspects of the care provided. The answers given by care receivers to these kinds of questions, however, are often ambiguous. Care receivers can express satisfaction despite results that others would judge as unsatisfactory or even bad because they deem a pleasant relationship with the care provider more important. Care receivers can also articulate dissatisfaction because they did not get what they wanted. Determining the meaning of expressions of satisfaction or dissatisfaction therefore requires a careful look into the case, and interpretation of and deliberation about these expressions. Although satisfaction, dissatisfaction and discomfort of care receivers should be taken seriously, the outcomes of patient satisfaction surveys are not a good measure. Care receivers, or their relatives, certainly have a voice in determining the quality of care and should be allowed to speak out. But the way to enable this, we think, is by perceiving them carefully, connecting with and attuning to them, and deliberating with them during the entire caring process itself.

In mainstream quality systems, care professionals are generally evaluated and judged according to whether they adhere to the prescribed system, and follow rules, guidelines, protocols and procedures. These systems, rules and so on originate in the wish to improve care, to share helpful knowledge, to make transferable good practice, and to prevent harm. However, over time they have begun to dominate, pushing into the background the question: ‘but is this, here and now, good care?’ This shift in emphasis has led to much improvement in the quality of care since the nineties of the last century but also to an increase in the administrative burden for professionals, which in turn has led for many of them to loss of motivation, burn-out, moral fatigue and so on. In addition, incidents with casualties still occur as well as not-so-good care. This has of course been noted in many forums, studies, reports, newspaper articles, documentaries and so on but the question remains of what went wrong and how it can be tackled.

Our point is that good care is not simply a matter of following rules, however helpful in general those rules may be, and nor is care meted out strictly according to rules necessarily good care. Many quality systems are unable to perceive ‘mismatches’: care given according to external rules without being attuned to the care receiver (Baart 2002, 2013; Baart and Steketee 2003; Goossensen et al. 2014). This is where practical wisdom comes in, because this is what is required to be both guided by rules and, at the same time, be well-attuned to the care receiver involved and find out what good care may be for them.

At the moment of writing, we are still in the middle of the Covid-19 crisis and it is too early to have a complete overview. What we see is quite ambiguous, showing at least two contradictory sides to every issue. On the one hand relational caring is easily put aside when social distancing is being used to fight a threat to the physical health of particular groups of people labelled as ‘vulnerable’, especially those living in institutionalised settings. But on the other hand, we also see a growing realisation that social distancing is detrimental to the physical and mental health of other groups of vulnerable people, particularly those living at home and deprived of their usual care, and that people are finding ways to be physically near them. Across the world, we see the criterion of safety becoming dominant, overruling all other quality criteria and shutting off political-ethical thinking. However, at the same time we also see people standing up for other quality criteria based on relationality, not only in regard to their family but also on a macro level, for instance in regard to refugees.

We see the consequences of how social forces have been operative, for example in the introduction of (limited) market competition and just-in-time production and delivery in healthcare, but we also see firms finding ways to help healthcare organisations by producing protective equipment for free or at cost price. We see care organisations being judged on whether they have followed rules, especially when mortality rates are well above average. But we also saw, when the pandemic hit the Netherlands, the Inspectorate and the Care Assessment Center putting their rules and systems aside and providing room for the practical wisdom of care professionals. In many countries around the world, we see variants of a total lockdown, based on distrust and repression of the population. In other countries, we see variants of an intelligent lockdown, built on trust and encouraging a sense of responsibility among the population within a guiding framework. We do not know yet which kind of lockdown will turn out to be most adequate, but the crisis raises questions about the kind of society we want to live in.

In this paper we present and elucidate a way of thinking about quality that can:

- do justice to relational caring, also in times of Covid-19;
- give voice to care receivers and those closely involved with them according to their concerns;
- perceive mismatches, without disregarding rules, guidelines, protocols and so on;
- appreciate the practical wisdom of care professionals in view of the uniqueness of each and every case.

At the same time, we present the CEMQUE model, a model to guide inquiry into and critically determine the quality of care received.

## Stagnating quality thinking

There is much excellent literature about quality, quality assurance, accountability for quality, strategies for improving quality and quality systems, also and especially in healthcare and social welfare (de Jonge et al. 2011). There are discussions, to mention just a few, about the significance of measuring patient satisfaction (Tzeng and Yin 2008), alternatives to the traditional ISO oriented approach (Harteloh 2003), the contribution of publishing performance data to the improvement of quality of care (Fung et al. 2008; Metcalfe et al. 2018), the impact of quality systems on the daily practice of (mental) healthcare (van Geffen 2019), the regulatory pressure (Banerjee and Armstrong 2015; van de Bovenkamp et al. 2020) and the administrative burden for professionals (Banerjee et al. 2015; de Veer et al. 2017; Woolhandler and Himmelstein 2014), the performance paradox (van Thiel and Leeuw 2002), the performativity of accounting (Drost et al. 2017) and indeed accountability itself as a ‘matter of care’ (Jerak-Zuiderent 2013; Ubels 2015). However in the practical implementation of quality thinking, including its theoretical foundation, and despite scholarly criticism, we see a lot of thinking that is stuck.^4^ We now mention four factors contributing to this stagnation.First, as soon as designers of quality systems start to think about quality in healthcare and social welfare, substantive thinking seems to disappear into the background. What care really is – a moral practice through and through that receives its guidance from the situation – and what social interventions actually serve – organising the political-ethical order of society – no longer receive attention. The important normative aspects of healthcare and social welfare are constantly concealed. If at all, moral issues are addressed as existential or psychological issues.

Second, quality thinking and the systems being built to guarantee quality seem irrevocably to end up with a heavily rigged set of checks and balances. According to Thomas Schmidt (2017), our fixation on quality means that we become entangled in all sorts of paradoxes (cf. van Thiel & Leeuw, 2002) and that we initiate mechanisms that we can no longer stop (cf. Shojania 2019). Quality policy is about an acceptable balance between what is good and what is not good at the same time. There is practically nothing to be found that is fully good and not at the same time, from a different perspective, not good.

Thirdly, in healthcare and social welfare, several logics and discourses are dominant that are not easily compatible with the ‘logic of care’ (Mol 2008), of which relationality is a key part. Quality policy often comes from the domain of the production of goods, serving the market, matching supply and demand, and binding customers to your product. This ‘logic of production’ does not fit care and nor does medical thinking, which is also dominant and not easily compatible with the logic of care. There is not only a difference in intentionality (cure versus care) but also in the underlying science, epistemology, conceptualisation, research methodology and normativity (cf. Mol 2008). In this paper we want to ‘think care from caring’ (Vosman 2014) and not from healing, solving problems, manufacturing or service providing.

The fourth factor refers to common ideas about organisation and management that often prove to be inconducive to solving the aforementioned problems. We need to consider the institutional embeddedness of the care offered: the style of management, the processes of policy formulation and, for example, all those routines, scripts, roles, conventions and expectations that take the obvious for granted. Such attention often falls outside the usual quality frameworks. Also, moral deliberations easily conform to what seems to be self-evident.

Thinking about quality should start with thinking about care. Care is not a set of separate care acts, but a ‘practice’, an ‘interplay of sayings, doings, undergoings and artefacts’, crossed by other practices and involving many different aspects and levels. When it comes to thinking about quality, these all need to be covered. As pointed out by care ethicist and political theorist Joan Tronto, good care is integrated care (Tronto 1993). Attention to quality is not an issue in itself but is interwoven with all kinds of learning and reflection processes (McPherson et al. 2001). Moreover, care is a complex practice in which care receivers and caregivers must deliberate to find out what good care is. Quality cannot be determined by general criteria but must be discovered locally per person and situation, and together with the person involved. Finally, care is a moral practice. Determining quality is impossible without considering moral issues, asking moral questions and making moral judgments, without disregarding the ambiguities of human life (Vosman 2018).

## Relational caring, quality awareness, and practical wisdom

Mainstream quality systems are hardly able to perceive and appreciate relational caring because they are oriented to ‘objective’ norms and values. This is generally the case although most care practitioners themselves will acknowledge that care is mainly ‘good’ in a particular context, in a specific situation, and at a determined moment in the ‘tangible’ life of a particular person. In this paper, we present an alternative approach, based on three pillars: (a) relationality or situatedness of care and, thus, of assessing quality; (b) the cultivated, self-regulating awareness of quality and (c) the practical wisdom of professional caregivers.

### Relational caring

In relational caring the source of action is the good that emerges within the relational network in which cared-for and carers find themselves and each other and interact with each other (cf. Habran and Battard 2019). Two intertwined concepts are therefore essential: relationality and finality, not as principles but rather as two focal points.

Relationality is about: who is participating, how are they positioned, and how do they position themselves and each other, what is happening in the interaction between them and what is the meaning thereof for them. In the weak sense of relationality, relationships are conceived of as merely conditional or instrumental to good care and not themselves as an integral part of good care. Essential to relationality in a strong sense is that what is happening, emerging and discovered in relationships between caregivers, care receivers and the people around them has real consequences for what is done and aimed at, and not only for how it is done. Relationality also has consequences for how the context, the situation and the persons within this situation are perceived.

Finality is concerned with what the practice is about, also from a historical perspective, and what participants actually are aiming at in what they do. Finality should not be understood as what people or organisations set as their particular goals. Essential to finality in a strong sense is that what is aimed at is not aimed at without considering the concrete relationships between care receivers, caregivers, the persons around them and their lifeworld, life course, et cetera. In fact, a good relationship is a good in itself.

These two focal points and how we conceive of them make our conception of relational caring a radical, and in this radicalism a quite unique, conception with comprehensive and far-reaching consequences. On the side of the professional, relational caring requires holding back, being sensitive and attentive to what happens in everyday life, allowing emerging goods to show themselves. It also requires managing one’s professional power and enduring one’s professional powerlessness, in order to prevent professional and bureaucratic perpetuation of the suffering and neediness of the other. Professional power should be exercised in the perspective of the ‘broken good’ and as ‘relationship-oriented, professionally loving and sensibly muddling through’ (Baart 2001, 2013; Schaftenaar et al. 2018).

### Cultivating quality awareness

Professional caregivers have been taught to follow rules, guidelines et cetera, and have learnt that they will become vulnerable to criticism if they deviate from those rules, guidelines et cetera. Consequently, they have become unfree in their perceiving, acting, reflecting and judging. Assessing relational caring, however, inevitably must be done (a) on the spot, (b) in the relationships between caregivers and care receivers and (c) momentarily. As care is a moral practice, assessing its quality is also a moral practice. This is a radically different approach from mainstream approaches.

What about public accountability? Measuring and presenting numbers is just one way of examining quality and accounting for good care. Usually this is done by producing a broad variety of quantitative performance data by which care organisations are compared. Numbers can be very informative but ‘have to be seen in relation to the ambiguities of the work and investments they entail’ (Jerak-Zuiderent and Bal 2011: 240). Other ways of examining and accounting are the narrative or hermeneutic approach and the phenomenological approach (Baart and Willeme 2010; Burhans 2008; Charon 2006; Vosselman 2014). In this paper, we focus on a fourth method: a developed and cultivated, (self-)reflective ‘quality awareness’, nourished by and expressed in perceiving, understanding, responding and accounting well (Baart 2014). The other methods of quality examination mentioned above can be used within this frame by bringing them into communication with each other informing and fuelling the ongoing inquiry into good care horizontally and vertically (cf. Sonja Jerak-Zuiderent’s concept of ‘generative accountability’, Jerak-Zuiderent 2013).

In order to carefully examine the quality of relational caring, one does not only need external tools; one must become a tool oneself, continuously and carefully inquiring into and in the concrete situation. This applies not only to the individual professional, but also to teams of professionals and the organisation as a whole. We propose that the cultivation of critical, vigilant quality awareness be made the core of quality policy.^5^ Permanent quality awareness maintains the connection between learning to perceive, to understand, to evaluate and to act. It looks at the entire process, including the person counting and accounting, and thus keeps together the different ways of looking at quality. Giving an account takes place at three levels: the internal level within the organisation including the care receiver, the internal level with third parties and the external level outside the organisation itself. Accounts are given first of all to the care receiver, on a continuous basis during the care given or proposed, and with an immediate response. Relational caring, examining quality and (horizontal and vertical) accounting are intertwined (den Bakker 2018).

Regardless of which learning and reflection practices are chosen, sooner or later people need a substantive idea of what constitutes good care or at least questions in that direction. In the next paragraph, we will present, step by step, a detailed model of good care that both nourishes quality awareness and makes it concrete.

### Practical wisdom of professional caregivers

In thinking about professionals and professionalism, scholars have moved from the classic professions of physicians, lawyers and clergymen to, first, technical-rational professionals with their evidence-based knowledge and practice, and then to normative-reflective professionals, aware of the different normativities intrinsic to their work. In response to the issue how to get from (formally, discursively and incorporeally) reflecting to acting, we developed the concept of practically wise professionalism. Practically wise professionals: (a) know what their profession is and what it is aimed at, and in concrete situations head for it; (b) accept that they must act, even if goals are contradictory, rules contradict each other and the outcome of acting is uncertain; (c) understand that complexity, dynamics and emergence belong to their profession and should not be unduly simplified or suppressed; (d) involve their entire personality, know their working style and realise which metaphorical positions they occupy; (e) conceive of rules and guidelines as indications of how to act based on the instructive experience of sensible colleagues in similar situations; (f) dare to take the leap to actually acting; and (g) respond to the question of accountability by telling the whole story from the inside out. In actual situations, practical wisdom implies making room for moral intuitions, moral imagination, interruption, finalising, deliberation and risking the leap to action (Bontemps-Hommen 2020; Bontemps-Hommen et al. 2019, 2020; Timmerman and Baart 2016)

## The care-ethical model of quality enquiry (CEMQUE)

Drawing on the conceptual foundations of relational caring, quality awareness and practical wisdom, this paragraph deals with the presentation and operationalisation of a detailed and innovative model. It was not developed on the basis of a definition or theory of quality, but by acknowledging that the quality of care that is relationally arranged, attuned and navigated, can only be determined locally. We developed our model based on (a) a strong philosophical and theoretical underpinning of what care actually is and (b) our experience in empirical and theoretical research, and validated and fine-tuned it in (c) training of (teams of) professionals and (d) discussions with professionals and organisations in healthcare and social welfare in a Quality Workshop held from 2012 till 2018. Our model is first of all a heuristic model that should help professionals and organisations to describe, assess and improve care, give an account of good care, assess existing quality frameworks and formulate quality policies. We have found it helpful in evaluating complaints and incident reports.

Before presenting our model, there are two decisions we must explain. The first is that we decided to take perspectives – of the care receiver, the caregiver, the organisation and society – and not relations as entries to the model. A pragmatic reason for that decision was that taking perspectives as an entry point is more attuned to how care receivers, professionals and organisations momentarily tend to think. A more fundamental reason is that in our conception of relational caring, connecting with and attuning to the care receiver and their lifeworld, life course and so on is essential. To connect with and attune to the other requires taking the perspective of the care receiver, i.e. this particular care receiver in his or her relationships with other people (Baart 2001). It is this perspective we wanted to be central in our model. Although we did not take relationships as entry points to our model, the different aspects of the model are elaborated in terms of social relations and are themselves related and correlated to each other. In what combinations, with what weight and how these relationships function in concrete cases, has to be discovered by the people involved using the model in their deliberation about good care.

**Fig. 1:**
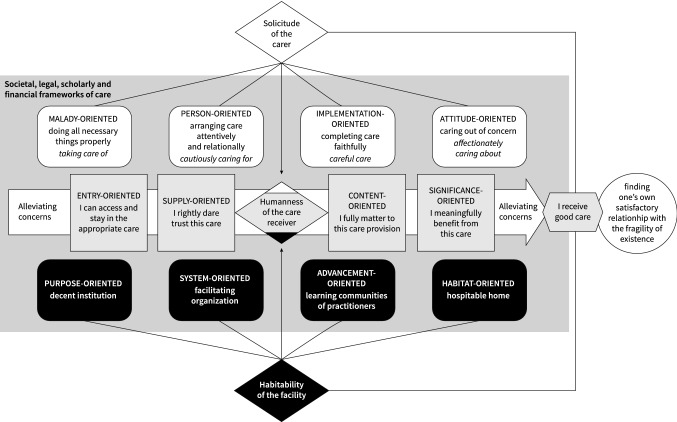
The Care-Ethical Model of Quality Enquiry (CEMQUE). (Retrieved, slightly adjusted and translated from Baart 2018 and graphically designed by Erik van Gameren.)

The second decision we must explain, was that we placed our idea of the ultimate goal of good care, its *telos*, as a focal point in the model. Continuing the previous discussion, good care must make a difference in the lives of care receivers. In the history of care ethics, care was seen as a response to the ‘moral injunction’ ‘not to turn away from someone in need’ (Gilligan and Attanucci 1988: 73) and the ‘purpose of caring’ in ‘the care-receivers’ experience of being supported and not left on their own’ (van Heijst 2011: 3). In the empirically grounded presence theory of Andries Baart, it was acknowledged that the good is unapproachable without the tragic, which is woven through everyday life as a complex structure. When nothing can be done, in terms of healing or problem solving, when life stagnates or collapses, one can always stay with the other (Baart 2001). The ultimate goal of good care, its *telos*, is to help the care receiver to find their own, more or less satisfactory relationship to the fragility, perilousness and transience of human existence: accepting, fighting, cursing, repairing, celebrating, undergoing or evading it (or a combination of these). Good care is care that helps the care receiver to realise their good. The care receiver’s good itself, however, lies beyond the scope and the responsibility of the caregiver and even the care system (as one can see in Fig. 1). What care can contribute is the alleviation of the care receiver’s concerns, though it can also aggravate those concerns. We will come back to the issue of ‘concerns’ below.

The Care-Ethical Model of Quality Enquiry (CEMQUE) consists of four normative layers or entries (see Fig. 1):

- humanness, from the perspective of the care receiver,
- solicitude, from the perspective of the professional caregiver,
- habitability, from the perspective of the care organisation and
- justice and decency, from the perspective of society.

The fourth entry to the model concerns the embedding of care in society with its societal care paradigms that determine the societal, legal, scholarly and financial frameworks of care.

In the following paragraphs, we elaborate on each of the four entries to the model. For each of them, we start with a ‘small philosophy’ of the keyword or overall criteria for quality at that level: humanness, solicitude, habitability, and justice. In these small philosophies we draw not only on the literature about the concept involved but also sketch out our own conceptual space. The four keywords represent (sets of) questions that drive the inquiry and at the same time keep it multi-dimensional and broad, rather than principles that should be applied and balanced against one another. Also, for each of the entries, the quality criteria at that level are clustered according to a qualitative procedure striving for maximum internal homogeneity and at the same time maximum external heterogeneity. It turned out that at each level the clustering resulted in four clusters, each under a heading that is not completely independent of theoretical thinking.

### The entry of the care receiver

Care receivers are central to the quality assessment of care (Amelung et al. 2017; Carey 2017; De Chesnay and Anderson 2019; Hewitt-Taylor 2015). In our model, they are implicated everywhere, but explicitly present in this first entry. In Fig. 1 this entry is represented by the middle row, with the wide arrow and the grey ‘buttons’. The idea of a button is that each ‘activates’ numerous questions and worksheets that inquire into that particular aspect of good care.^6^ Because of the complexity of care, each button also activates other buttons.

The ultimate goal of good care is to help the care receiver find their own satisfactory relationship to the fragility of existence. What care can contribute is the alleviation of the care receiver’s concerns. That is what the wide arrow at the centre is about. People who call on care, help and support, may expect the professional to do the work, but they must do the work too – sometimes considerable work. Besides the issue that prompted them to call on care, help or support, they often have other issues they struggle with. We call them ‘concerns’. In general, concerns have four aspects in common: they indicate a certain trouble, have a personal character, impose themselves with urgency and require an effort from the patient or client. Care receivers’ concerns can be taken away and alleviated, but also created and aggravated by caregivers and their responses to what they perceive as the care receiver’s concerns (Olthuis et al. 2014). The caregiver must come close to and seek a relationship with the care receiver in order to properly perceive their real concerns and give an adequate, fitting response.

The overall quality criterion at the level of the care receiver is their, what we term, ‘humanness’. It is not quality of life, or autonomy. With the term humanness, we enter a complex field of meanings, definitions and usages from different disciplines. Because we are interested in those aspects of humanness that can be affected by care, we deem four elaborations of the concept relevant. First, humanness is due to every person, does not have to be earned and cannot expire. Anyone who tramples on the humanness of somebody else will destroy something that we collectively find precious. Humanness is often a counterfactual concept. Second, human existence takes place in extensive networks of interdependencies and reciprocities, including ‘relational autonomy’, a conception of autonomy that is more social than personal, more diachronic than momentaneous, more a matter of degree in various aspects than of yes or no regarding the rational capacity to make decisions regarding one’s own life. Every person is part of the human family; humanness is fellow humanness is citizenship. Third, humanness is also connected to general and deep-seated human desires, including the desire for recognition. These desires must be fulfilled in one way or another if people are to be ‘human’. Customization is required in this fulfilment. Humanness in healthcare involves the demand not to add to suffering and to find the right balance between general supply and unique focus (van Heijst 2011). Humanness as an umbrella concept also introduces the idea of personal identity. Fourth, because humanness is connected to what is precious and one therefore prefers not to lose it, people are vulnerable. Vulnerability is inherent in humanness. There are many types of vulnerability. You can distinguish between intrinsic and extrinsic vulnerability, and between potential and current vulnerability, but in many cases these distinctions fade away. You can also become more vulnerable through receiving care. The bottom line in all these elaborations is: do not increase or prolong suffering, do not expropriate the life of the other, and do not refuse access to health and/or social care.

The quality criteria pertaining to the level of the care receiver can be clustered into four categories according to their orientation toward the entry to, the supply of, the content of or the significance of the care offered or provided. When the entry-oriented criteria – the first category – are met, the care receiver could say: ‘I can access and stay in the appropriate care’. Good care is structurally, relationally and culturally accessible, findable and attainable. When the supply-oriented criteria – the second category – are met, the care receiver may say: ‘I rightly dare trust this care’. Good care is transparent, sound and reliable. Three issues are relevant: what does care do for me? What does care ask from me? How does care protect me, especially my privacy? When the content-oriented quality criteria – the third category – are met, the care receiver may say: ‘I fully matter to this care provision’. Good care is honourable, shared and steerable. This steerability is related to recognition: relational recognition of the unicity of the care receiver, discursive recognition of their knowledge, and political recognition of their position. When the significance-oriented quality criteria – the fourth category – are met, the care receiver could say: ‘I meaningfully benefit from this care’. Good care is appropriate – that is, helpful, tolerable, acceptable and fitting to the care receiver’s life – and beneficial – helpful in finding one’s own satisfactory relationship to the fragility of human existence. Is this care focused on allowing you to live your life in whatever modality that you can call yours?

In the Covid-19 pandemic the fragility of existence comes to the fore. How to deal with this fragility – for example when, lying with Covid-19 in an IC-unit, one must be put to sleep – is of more concern to the care receiver and their family than quality of life. The concerns of care receivers and their family become part of the equation. In nursing homes, it turns out that in many cases the actual concerns of residents and their families are not known very well. In March 2020, 15 staff members of the San Jerónimo nursing home in Estella, Spain, decided to lock themselves up with their 62 residents. They stayed together for 35 days, 24 h a day. None of the residents and staff became infected. This also had an effect on the the staff’s understanding; as the director later explained: “We understood better what the world of our elderly looks like”.^7^ Because of Covid-19 itself but also because of the measures taken, the concerns of residents and their family broadened and moved towards more existential questions. Dealing with the ban on visits by more than two people to your elderly mother with dementia in a nursing home, while you have two young children and are simply referred to the care assistants for ‘questions’, can be a major struggle. Surveys do not easily capture such concerns (cf. Wammes et al. 2020). In general, the residents’ families are reduced to ‘visitors’ and their care for their family member discounted (Frederiks et al. 2020). On the other hand, we also see care receivers having an easier life and finding more peace of mind because everyone must stay at home. It remains to be seen, however, whether having more peace and less conflict because no one can visit you and your fellow residents, indicates more quality of life. The model urges inspection of the impact of the measures on access, trust, acknowledgement and experienced benefit. Each of the four buttons ‘lights up’. It becomes clear that each of the various buttons in itself represents an area of tension. And that these different tensions influence each other.

### The entry of the caregiver

The professional caregiver is envisioned in the second entry to the model, in Fig. 1 represented by the top row, with the white buttons.

The overall quality criterion on the level of the caregiver is their ‘solicitude’, not their expertise, competence or ability to intervene, repair or cure. Solicitude is a relational concept: becoming involved with another person who is in need. It is also a reciprocal concept: involvement in another person’s suffering that has affected the caregiver. Solicitude includes a bandwidth of motivation: from worrying at a distance (worrying about) to feeling called on to act (caring about). This raises an interesting dialectic: the practising of solicitude is itself also a vehicle for developing the idea of how solicitude can best be practised. Solicitude is not just an emotion, but also a will that must both become practical and be sustained. It is also an evaluative term – something or somebody else is in miserable circumstances – and it entails an element of moral imagination – refraining from doing something will lead to more misery. Solicitude is set against the background of social precarity (Baart 2021).

The quality criteria pertaining to the level of the caregiver can also be clustered into four categories according to their orientation towards the malady or the person of the care receiver, and towards the implementation of the care or the attitude of the caregiver. The malady-oriented criteria pertain to doing all necessary things properly, fairly, safely, competently, in a timely fashion, transparently et cetera (cf. Donabedian 2003; Hughes 2008; Moulin 2002). They ask whether what is happening in a specific situation is good care, given the available discipline-specific knowledge regarding the person’s malady, defect, trauma, need, damage or injury and the available remedies. Given the situation, the malady and the available remedies, what goals are we pursuing in a professional and practical manner? Good care means adequately taking care. What is assessed by these criteria is professional competence.

The person-oriented criteria pertain to arranging care attentively and relationally (Baart 2004a; Klaver 2016). They ask whether what is happening in a specific situation is good care, given the available knowledge about the suffering person’s lifeworld, life course, longing and concerns and, in that context, about the concrete meaning of this malady for this person? Are we adequately connecting with and attuning to their lifeworld, life course et cetera? Given their lifeworld, life course, longing and concerns and the significance of the malady, what goals are we pursuing for this person in an attentive and practical manner? Good care means cautiously caring for. What is assessed by these criteria is attentiveness and dedication (cf. Schaufeli 2013).

The implementation-oriented criteria pertain to faithfully adjusting and completing care. They ask questions about starting, giving and implementing care, about keeping on course, adjusting and completing care. Are we taking responsibility, even where those who should do so in fact do not? Good care is faithfully completing care. What is assessed by these criteria is responsibility.

The attitude-oriented criteria pertain to caring out of engagement. They raise the question of what form engagement should take in relation to how the client is being treated and which emotions are being shared? Is this being done in a way that both the person cared for and the caring professional benefit from it? Are professional carers emotionally involved in an appropriate, controlled and managed fashion? Are they preventing themselves from having a ‘burn-out’? Self-care is part of good care. What is assessed with these criteria is engagement, being involved with the other person ‘with distance’ but not ‘at a distance’ (Stoopendaal 2008).

Good care is integrated care (Tronto 1993), i.e. care oriented towards both malady and person, both implementation and attitude. In the measures taken in response to the Covid-19 pandemic we see an emphasis on the malady and implementation-oriented aspects of care. Other aspects of care, oriented towards the person of the care receiver or the attitude of the caregiver, do not get the attention they deserve or, in the case of person-oriented aspects, are delegated to staff from other disciplines like the psychologist or spiritual counsellor. However understandable this may be because of what patients are undergoing and caregivers are struggling with, the longer these measures last the more problematic they become. We see caregivers asking for clarity about the rules and managers who do not trust their employees to comply with them. Most tragically, caregivers are sometimes unable to complete their care because a resident must go to a Covid-19 ward or a resident’s corpse must be removed immediately after death. On the other hand, we also see more emphasis on person-oriented aspects of care among caregivers and increased engagement. We see solidarity between them, and creativity within the rules. We see different ways of providing ‘care from-a-distance’ being tested, promoted and developed. However understandable and even necessary at this moment, the question remains of how this relates to the necessity for good care of being physically near to and touched by the other person. We also see policy makers realising that the core of good care is relationality, but not knowing how to facilitate it. The issue of self-care is particularly relevant in the trade-off between safety and dedication. Each of the four buttons lights up and becomes a discussion issue. The case of De Riethorst nursing home provides several examples of this.

### The entry of the care facility

The care facility is envisioned in the third entry – in Fig. 1 represented by the bottom row, with the black buttons.

The overall quality criterion on the level of the care facility is ‘habitability’, not hospitality or safeguarding. Habitability is a relational concept. It refers to an organisation, finalised as an institution, that offers a space to live, work and entertain an identity to those who take refuge there and those who must be there because of their work. It is also a political concept: what room are we prepared to make for our guests, who are also strangers and often a kind of rivals, enemies or intruders? Is what is habitable for one perhaps excluding another? First of all, entering care entails crossing a border, going from an ‘outside’ to an ‘inside’ that has its own objective, regulations and logic. By immediately putting the emphasis on habitability when it comes to thinking about the organisation of care, the precariousness for the care receiver to enter another space and to put themselves under a different regime comes to the fore. Habitability can include bringing into a safe place and, in that place, preserving or sheltering. Sometimes habitability is contrasted with being uprooted.

Secondly, in a habitable resort people receiving care are almost always guests. They live in someone else’s world. The temporary sheltered place can easily become a ‘total institution’. Erving Goffman describes the terrible ‘mortification’ of residents who have become embedded in a system of adaptation, gaining privileges and dodging (Goffman 1970). If care offers too much comfort and too little challenge, it can happen that care receivers give in, become dependent and ultimately lose themselves. The ‘stranger’ is aligned with the standard citizen and normal person, and thereby alienated from their inner world and lifeworld. Habitability is only habitability if it can control the totalising danger of care giving. Thirdly, habitability accommodates hospitality, disconnecting it from the feeling of ‘being at home’ that can also be accompanied by the exclusion of other people. We regard hospitality as the organisation and arrangement of an open reception of both care receivers and caregivers. Finally, habitability is also a keyword for a societal order in which potential caregivers and potential care receivers are already engaged in each other.

The quality criteria pertaining to the level of the care facility can also be clustered into four categories, according to their orientation towards purpose, system, advancement or habitat. Corresponding to these four categories, there are four materialisations or bearers of facilitating good care: the institution, the organisation as facilitator, the learning communities of practitioners and the organisation as offering a home. The normative colouring comes from the ‘decent institution’, finalised well. The articulation of the finality of the institution and maintaining it is a discursive achievement, the result of a deliberation of all involved in the institution. This is being fed directly, by the learning communities of practitioners, and indirectly, through the learning communities, by the organisation as facilitator and as offering a home. As opposed to similar approaches to caring organisations such as proposed by Moore and Beadle (2006), our approach is consistently conceptualised as relational. We conceptualise an organisation as a collection of practices that need to be connected. The concept of ‘decent institution’ is central. Organisations have an underlying goal (*telos*) and must contribute to a decent society. We conceptualise good care as solicitude. The four criteria bearers of habitability are dynamic practices that are connected. Habitability constitutes an embedding of solicitude, and solicitude makes demands on habitability.

The purpose-oriented quality criteria are oriented towards a decent institution that discursively is finalised and finalises. A care organisation must be able to find the right balance between, on the one hand, the finality or *telos* of the institution (what are health and social care ultimately about?), the political-public rules of implementation (governance) and its own actual possibilities and limitations. This means compromising but then ‘decently’ (Margalit 1996). Finding and (for the time being) formulating its *telos* (its *raison d’être*, core business, internal goods, scope, inner destiny, tenet) is a difficult process in which three dominant practices – of the professional, the management and the board – all need to be involved. A supporting environment should also be sought.

The system-oriented quality criteria are oriented towards a facilitating organisation that organises its processes in a reticent way and provides a habitable house for its employees. Does the organisation provide room for its professionals in such a way that there is space for their knowledge and understanding, for them to search out what good care is, and learn to take responsibility and to manage themselves properly? This requires organising and managing along modest lines: simple structures, trust in craftsmanship, only rules that support, managers as ‘heat shields’ who shield professionals from incentives from above that do not help.

The advancement-oriented quality criteria are oriented towards creating learning communities that cultivate a shared quality awareness. This also implies facilitating vertically: consultation, feedback, deliberation, support, normative reflectivity, joint experiments, learning communities.

The habitat-oriented quality criteria are oriented towards offering a hospitable environment that offers a sense of being at home. Habitability implies compassion at the institutional level for both clients and staff. This translates into an organisational culture and practical, physical and organisational conditions and provisions.

During the Covid-19 pandemic, care facilities are under great pressure. The purpose of care becomes (re)formulated in terms of ‘safety first’: for patients and clients, for professionals, for society, for the country. There is not much and not very substantial thinking and deliberation about finality. Under the surface lie questions such as: which patients and clients, which professionals, which societies, which countries, and which not? In the name of safety, quality awareness comes under pressure or is even temporarily deactivated. Guidelines and regulations from the government or sector organisations are implemented unquestioningly and often more rigidly than the guidelines or regulations themselves prescribe. Learning communities suffer from measures of social distancing and do get cancelled because acting takes precedence over reflecting. Nursing homes take on the form of a closed bastion instead of a hospitable home, for both clients and their families as well as professionals and volunteers. What kind of support do professionals receive from their organisation when they *reflectively* make decisions? How reticent and purpose-oriented are organisation and management able to stay during the crisis? How free or restricted becomes what can be aimed at? On the other hand, the relevance and importance of a well-organised facilitation of professional caregivers come to the fore too. We also see a greater need to ask the finality question. In some contexts, the management succeeds in organising care in a reticent way and leaves more space for improvisation and practical wisdom. De Riethorst nursing home managed to continue reasoning from their finality, articulated as being a home for their residents and facilitating their freedom of movement. Again, each of the four buttons lights up.

### The entry of society with its societal, legal, scholarly and financial frameworks of care

Society with its care paradigms is envisioned in the fourth entry to the model as represented in Fig. 1 by the grey plane, the background to the three rows representing the other entries. We call it the ‘copper plate’, as copper has the capacity to conduct very well. What counts as good care is to a large extent determined, made possible and limited by powerful forces in the background, which influence relational caring but can hardly be seen by or manipulated by care receivers, caregivers and care organisations. The political ideal of self-reliance and participation, the paradigm shift in the way people with intellectual disabilities are perceived, from patient to citizen, and the system of financing health and social care are examples of these hidden forces. Ordinary quality systems can have little influence on these forces because such systems themselves are embedded in these frameworks. A political-ethical orientation of thinking about quality is much needed.

This entry involves continuous and systematic thinking about whether the care provided contributes to ‘the good life with and for others in just institutions’ (Ricoeur 1992) ‘in a decent society’ (Margalit 1996). ‘The good life’ also implies the ability to live in a satisfactory relationship to one’s own fragility. ‘With and for others’ entails a double recognition: I must acknowledge myself (*soi-même*), but also the others (*autrui*) who are just like me. ‘In just institutions’ expresses the idea that the good life takes place in an orderly society, in which institutions make living together possible, but also confine it. Justice also includes recognition. A decent society is a society in which people are not humiliated by its institutions.

For now, we confine ourselves to four categories of systemic frameworks of care. Societal and cultural conventions and hypes determine who and what is deemed worth caring for and who is fit to take care of themselves. The legal determination of rights and obligations, tasks and accountabilities determine who can claim care and who should provide this care. The scholarly and scientific paradigms determine what knowledge and whose knowledge counts and should be applied, and what room is left for alternative modes of knowing and moral considerations. The system of financing healthcare and social welfare determines what care is available and attainable, and under what conditions.

During the Covid-19 crisis, we see the copper plate come to the fore, in the first place in the far-reaching restrictive measures issued by the government in the name of safety. Although they are independent of the government and in spite of how their thinking about quality had developed during the previous decade, the boards of nursing homes implemented these measures without questioning them (den Uijl et al. 2020). Nursing homes restricted their residents’ freedom of movement for a long time and sometimes drastically, often on the wrong legal basis or a questionable interpretation of the law (Frederiks et al. 2020). We also see the copper plate functioning in the shortage of IC beds and nurses, masks and tests in the first months of the ‘intelligent lockdown’ in the Netherlands after decades of introducing and promoting free market and just-in-time production and delivery in healthcare. In these first months, nursing homes were provided with personal protective equipment much later than the hospitals. Some commentators have interpreted that as an expression of ageism. Another example of how the copper plate comes to the fore is the dominance of medical thinking in the policy for controlling the Sars-CoV-2 virus, often overruling the logic of care. On the other hand, competition is sometimes suspended in favour of cooperation and mutual engagement. Patents are cancelled in favour of open knowledge sharing. There is so much funding for developing a vaccine that it is being made available to the public far more quickly than would normally be the case. And we also see a public re-evaluation of professions such as those of nurses, care assistants and general practitioners. The first signs are already there, however, that this re-evaluation will not have enduring consequences. And that the rich countries will not help the poor countries to vaccinate their population first although that would be the most effective from a world perspective.^8^

## Discussion

In this paper, we presented the CEMQUE model and put it to the test of residential care for older people in the Netherlands during the Covid-19 pandemic. We did it based on our own observations, media coverage and scholarly publications by others. In Corona times however, it is difficult to access nursing homes and deliberate with workers about the dilemmas they face and the choices they make. This is a serious limitation on how we are used to working: in close connection with caregivers and care organisations themselves. Another limitation, of course, is that we are still in the middle of the crisis and the final outcomes are still unknown. In the Netherlands, vaccination started on January, 6th, starting with caregivers of Corona patients, but it will take some time before the vaccination coverage is high enough to make a difference, first in the nursing homes and then in society in general. However, we were able to make plausible that the CEMQUE model is indeed helpful and revealing, allowing us to perceive, make explicit, question, evaluate and give an account of good care in healthcare and social welfare in general, and also during the Covid-19 crisis. All four levels in the model turned out to be relevant, and on each level, each of the four buttons mattered. Also, it became clear that each of the various buttons in itself represents an area of tension. And that these different tensions influence each other.

In the CEMQUE model, the *telos* of good care is to help the care receiver to find their own, more or less satisfactory relationship to the fragility, perilousness and transience of human existence. The Covid-19 pandemic has shown that it is indeed a much more adequate articulation of the purpose of good care than adding quality to the patient or client’s life. When dying in loneliness and agony, good care has nothing to do with quality of life. Quality of life suggests something objective that (a) can be accomplished, (b) can be determined to be good from the outside, and (c) is without a doubt good. We question all three of these statements: (a) what is good in someone else’s life can be provoked or promoted, but eventually must be found or discovered by the person herself, (b) determining that something is good in someone’s life is an evaluative act by the person herself in that particular situation, and (c) the good is always and inevitably a ‘broken good’, mixed with what is not so good or even evil. Good care is care that helps the care receiver to realise their good. The care receiver’s good itself, however, lies beyond the scope and the responsibility of the caregiver and the care system. Healthcare and social welfare can be helpful, but should not pretend, as Ivan Illich already made clear, to be able to take away the necessity to ‘come to terms with pain, impairment, and death’ (Illich 1975: 90). This raises the question of why it is nevertheless most often ‘quality of life’ that is invoked when someone wants to criticise the overwhelming focus on healing, mending and repairing – and safety.

What care can contribute is the alleviation of the care receiver’s concerns and struggles, though it can also aggravate these. We have seen the concerns of care receivers being aggravated and amplified by the rigorous policy of ‘social distancing’ and by the banning of visits. We think that it is more coherent with thinking in terms of relational caring to talk about ‘concerns’ and engage in an inquiry to discover the actual concerns of care receivers rather than talking about ‘rights’ and trying to balance them against each other. For example, the right to autonomy and self-determination against the right to be protected against illness and death.

In the case of residential care for older people, the visiting ban also aggravated the concerns of the relatives, transforming them from informal caregivers into ‘visitors’. We also became aware of the concerns of the caregivers and how they were aggravated, particularly when families who were critical about the measures announced were referred for ‘questions’ to the caregivers caring on a daily basis for their family member.

Finally, the social experiment that the Covid-19 measures actually constitute, raises questions regarding the placement of quality awareness at the centre of the model. We see, on the one hand, that relying on it is mistrusted and associated with major risks. But we also see, on the other hand, that professionally loving and sensibly muddling through is practised and promoted on different levels in society. Muddling through, however, activates and requires quality awareness. We can now learn more about how to give it room, maintain and nourish it. It requires training, education, trust, a specific leadership, and the positioning of people as citizens in a political-ethical sense of the word: each one of them as a constituent of the sovereign.

## Conclusion

Although it is still too early to draw far-reaching or definite conclusions, we believe the Care-Ethical Model of Quality Enquiry (CEMQUE) passed the test of the Covid-19 pandemic, even better than mainstream quality systems. It is indeed helpful and revealing by perceiving, organising, making explicit, questioning, evaluating, promoting and giving an account of good care in healthcare and social welfare in general, during this Covid-19 crisis as well as in post-Corona times. Its strengths are the three pillars (relational caring, cultivating quality awareness, and practical wisdom), its comprehensiveness and coherence (of the four entries), the centrality of the concept of concerns in relation to the fragility of existence, and the inclusion of the political-(care-)ethical dimension. In deliberations with professionals, the CEMQUE model helps to see a larger margin of manoeuvre than is often thought possible. It draws attention to aspects that may be temporarily suppressed, but even in times of crisis deserve much more explicit attention if one wants to provide good care.

## Footnotes

1 According to the Coronavirus Resource Center of the John Hopkins University: https://coronavirus.jhu.edu/map.html (accessed 27 May 2020 and 11 January 2021).

2 We are grateful to Albert Banerjee and the two anonymous reviewers for their comments, criticisms, and suggestions.

3 The following case description was made on the basis of publicly accessible sources, listed by the care organisation involved itself: https://www.mijzo.nl/mijzo-in-de-media (accessed 15 December 2020).

4 We did not make a complete international comparison of the major quality reporting systems. Being familiar with the international literature in diverse disciplines such as ethics, philosophy, sociology and political science (Baart 2018), we focussed on quality policies and quality systems in healthcare and social welfare in the Netherlands. We consider the Netherlands an exemplary case due to its historically conditioned, international orientation, and receptivity to management and market thinking (de Vries 2007; Dwarswaard et al. 2011). At the intersection of German, French and Anglo-Saxon approaches to quality, the deficiencies of the individual approaches are sharply exposed, the discussions are diverse and rich, and the difficulties these approaches raise become sharply apparent.

5 The term ‘quality awareness’ originates from engineering and manufacturing (Crosby 1979) and was, at the suggestion of Andries Baart, adopted by ActiZ in the title of a two-year work programme (2012–2014) in which he participated (Ubels  2015).

6 Worksheets, in Dutch, can be found at http://qr.presentie.nl/link.php?qrlink=1g7sj3s (accessed 11 May 2020).

7 See https://nos.nl/artikel/2362124-hoe-het-coronavirus-volledig-buiten-dit-spaanse-bejaardenhuis-bleef.html (accessed 28 December 2020).

8 See https://www.nytimes.com/2020/12/15/world/rich-countries-have-first-dibs-on-vaccines-while-poor-nations-struggle-to-get-enough.html (accessed 28 December 2020).
